# Pasteur and His Methods

**Published:** 1887-01

**Authors:** 


### *PASTEUR AND HIS METHODS.*

The fame of Pasteur is rapidly declining. In his own country, now, many of the journals, both medical and secular, are publishing the numerous of his methods and even insinuating that the inoculation of antirabic virus is even dangerous to life. Among the prominent critics of Pasteur is Dr. Lutand, editor of *Le Journal de Medicine de Paris*, one of the best of French medical periodicals. The number of this journal for Oct. 31st, is largely made up of criticisms on the Pasteurian method and its advocates. The first article entitled "Pastoriana," is a violent attack upon Pasteur for advising a provincial governor, who consulted him about numerous cattle bitten by a rabid dog, to have the cattle slaughtered, saying that the quality of the meat was not impaired by the rabic virus. Even the literary style of Pasteur's letter is criticised and the distinguished savour is advised to betake himself to the primary school for elementary instruction in French grammar. In another article the statistics published by Pasteur are ridiculed. It is claimed that 2,500 persons bitten by rabid animals have been treated, with forty deaths. The extensive advertising which the distinguished scientist has received has induced people, especially the wealthy, to seek his laboratory for treatment, and it is extremely doubtful if many of these frightened individuals were really bitten by rabid animals. An article, entitled, "Pasteurian Necrology," is published from week to week, giving an account of the deaths occurring after treatment. Nine occurred in the week ending Oct. 31st, alone; two in France, one in England, three in Russia and three in Spain. Of the Spanish patients three out of four, bitten by the same dog, died. The Spanish Governor telegraphed Pasteur, informing him of the failure of his method and demanding an explanation. He received the following laconic reply: "I don't understand it all." Even the associates of Pasteur are beginning to recognize the uselessness of their methods, and now announce that they will soon produce a more perfected method of inoculation.

